# Response of the germinable soil seed bank of temperate *Leymus chinensis* meadows to mowing regimes

**DOI:** 10.3389/fpls.2024.1508711

**Published:** 2025-01-14

**Authors:** Zhitao Zhang, Tianqi Yu, Xiaoping Xin, Hongmei Liu, Shijie Lv, Zhijun Wei, Guodong Han, Ruirui Yan

**Affiliations:** ^1^ College of Grassland Science, Inner Mongolia Agricultural University, Hohhot, China; ^2^ Chinese Academy of Agricultural Sciences, State Key Laboratory of Efficient Utilization of Arid and Semiarid Arable Land in Northern China/Institute of Agricultural Resources and Regional Planning, Beijing, China; ^3^ Inner Mongolia Academy of Forestry Sciences, Hohhot, China

**Keywords:** mowing system, temperate *Leymus chinensis* meadow steppe, germinable soil seed bank, sustainable development, perennial grass, upper-growth grasses

## Abstract

Mowing is a primary practice in temperate *L. chinensis* meadows, which are severely degraded due to frequent mowing, overgrazing, and other factors, necessitating restoration and sustainable management. The natural recovery of these grasslands hinges on their germinable soil seed banks, which form the basis for future productivity. Thus, germinable soil seed banks are critical for restoring overexploited meadows. In this study, we conducted germination experiments on 135 soil samples from various depths to comprehensively analyze the germinable seed bank under different mowing regimes. The main results were as follows: (1) the germinable soil seed bank density decreased significantly with a mowing event per year (C1), and the number of perennial grass seeds and upper grass seeds also decreased under the mowing event per year; (2) the size of the germinable soil seed bank increased under the other mowing regimes (control area without mowing or grazing, CK; mowing event every 2 years, C2; mowing event every 3 years, C3; and mowing event every six years, C6) relative to that under once-a-year mowing. With increasing soil depth, the number of germinable soil seeds decreased significantly. Most of the seeds in the germinable soil seed banks were distributed in the 0–2 cm soil layer, accounting for approximately 80% of the total, and at depths of 5–10 cm, the number of seeds of upper grasses was greater than that of perennial grasses. (3). During the mowing event each year, the seed bank of germinable soil seeds significantly decreased. Mowing every 2 years provides a one-year interval for natural vegetation growth, allowing for greater retention of seeds in the germinable soil seed bank. Mowing every 6 years significantly reduces the disturbance frequency, providing ample time for plant reproduction and resulting in the accumulation of germinable seeds in the soil.

## 1 Introduction

Temperate *Leymus chinensis* meadow steppes are typical grassland ecosystems in Central Asia and have rich biodiversity and important ecological functions (Chesson and Warner, 1981). Germinable soil seed banks are vital for plant population reproduction and ecosystem resilience and play crucial roles in maintaining species diversity and ecological balance; they serve as a resource for vegetation renewal and a strategy to compensate for disturbances in plant communities (Dullinger and Hülber, 2011), and they play important roles in maintaining the stability of grassland ecosystems (Godfree et al., 2011).

Mowing is one of the main grassland utilization patterns in temperate *L. chinensis* meadows. The germinable soil seed bank contains seeds of a variety of plants that can germinate under suitable conditions, compensate for aboveground vegetation, and maintain the diversity of the community. Therefore, studying the effects of different mowing systems on the germinable soil seed bank to alleviate pressure from grassland degradation is highly important. Sun (2021) reported that mowing at a residual height of 2 cm significantly reduced the biomass of perennial grasses. Conversely, maintaining a residual height of 8 cm did not significantly differ in terms of grassland productivity compared with unmown plots and increased ecosystem species diversity. The author further emphasized that the optimal residual height should be chosen on the basis of local environmental factors in different regions (Sun et al., 2021)., Additionally, several scholars have demonstrated that mowing regimes can reduce the number of genera and individual density of soil nematodes, as well as decrease soil organic carbon and total nitrogen levels (Wu et al., 2007; Yuan et al., 2011). According to relevant studies, appropriate mowing systems not only increase species richness in aboveground plants (Diaz, 2007) but also increase the stability of plant communities (Yang et al., 2012); however, high-intensity and frequent mowing causes grassland patching and gradual degradation. Yan et al. (2021) reported a 25.01% reduction in *Leymus chinensis* yield after three consecutive annual mowing events in the temperate *Leymus chinensis* meadow steppe (natural hayfield), underscoring the impact of annual mowing on productivity. Notably, a 74.5% yield decline was observed after five consecutive annual mowings, highlighting the exacerbating effect of this management practice on yield reduction (Yan et al., 2021). These findings indicate that excessive mowing significantly affects ecological restoration and rational resource utilization in grasslands. In response to grassland degradation caused by excessive grazing intensity, scholars have proposed measures such as reducing grazing intensity and implementing grazing bans (fencing). In scenarios where the efficacy of germinable soil seed banks diminishes, strategies including no-till replanting, artificial sowing, fertilization, soil preparation, and revegetation across various ecosystems are recommended (Harris, 2010; Jin-Sheng et al., 2020). However, the potential role of persistent germinable seed banks in soils is increasingly prominent in restoring degraded grasslands to their original natural states (Guo et al., 2023).

Germinable soil seed banks consist of collections of dormant and semidormant seeds that are present on the soil surface and in the soil; these seeds play a key role in maintaining ecosystem health and diversity (Sloey and Hester, 2019) and harbor seeds that are independent of the parent and have the ability to develop into independent plants (Tóth et al., 2022). The germinable soil seed bank exhibits “succession memory,” containing not only the seeds of current aboveground plants but also those from earlier stages of succession in the region, as well as seeds of plant populations or new species introduced by animals. These elements play crucial roles in guiding future community structure and succession. Consequently, germinable soil seed banks are pivotal in the restoration of grassland ecosystems (Shiferaw et al., 2018; Wang et al., 2020). Many studies have been conducted on the effects of mowing on plant communities in terms of species composition, biomass formation and nutrient cycling (Maron and Jefferies, 2001; Moinardeau et al., 2019; Zhang et al., 2023). In addition, under nitrogen deposition, different mowing regimes may promote the decomposition of litter by some microorganisms, increase soil fertility, and provide suitable conditions for germinable seeds (Ya-Jing et al., 2004; Liu et al., 2018). There are few studies on soil seed banks in different soil layers through various mowing systems in natural grasslands, especially seed banks in temperate *L. chinensis* meadow grasslands subjected to mowing regimes. In particular, the mechanisms by which long-term continuous mowing impacts germinable soil seed banks, especially the germinable seed banks of dominant perennial grasses and upper grasses (upper grasses are characterized by reproductive shoots and long vegetative shoots, with a relatively large proportion of leaves distributed in the upper part of the plant community and relatively taller plants) in grasslands, have not been fully studied. In our consensus on the study of the germinable soil seed bank, most of the seeds are found in the 0–5 cm soil layer. Previous studies have shown that as depth increases, seed density and the number of germinable seeds decrease significantly (Shiferaw et al., 2018; Tuthill et al., 2023). Soil can be divided into 0–2 cm, 2–5 cm, and 5–10 cm layers, and three questions can be addressed: (1) Analyze how the germinable seed banks in soils at different depths change under different mowing regimes. (2) What is the vertical distribution of seeds in the germinable soil seed bank, and in which soil layer are the seeds mainly distributed? (3) How do perennial grass seed banks and upper grass seed banks respond to different mowing regimes?

## 2 Materials and methods

### 2.1 Overview of the study area and experimental design

In this study, a long-term mowing experiment was performed in the *L. chinensis* meadow grassland of the National Field Scientific Observation and Experimental State of the Hulunbuir Grassland Ecosystem in Inner Mongolia, which is located 10 km east of the Sheertala Breeding Farm in Hulunbuir city, Inner Mongolia, China, with geographical locations of N49°33′ and E120°05′, an altitude of 603.0 m to 776.6 m, and an annual precipitation of 351 mm. The landform types at the experimental site can be divided into low hills, high flatlands, low flatlands and river flats. The soil type in the experimental area was dark chestnut calcareous soil, and the vegetation can be classified as a zonal meadow *L. chinensis* community. The dominant species in the plant community were *Leymus chinensis*, and the subdominant species were *Cleistogenes squarrosa*, *Pulsatilla turczaninovi*i, and *Stipa baicalensis*. The common species were *Adenophora stenanthina*, *Artemisia tanacetifolia L*., *Allium bidentatum*, and, occasionally, *Heteropappus altaicus*. In this study, the species identified through germination experiments were *Leymus chinensis, Stipa baicalensis, Artemisia tanacetifolia, Cleistogenes squarrosa, Adenophora stenanthina, Thalictrum squarrosum, Astragalus melilotoides, Heteropappus altaicus, Potentilla nudicaulis*, and *Pulsatilla turczaninovii* (**Table 1**).

**Table 1:** Characteristics of the taxonomic composition of the germinable soil seed Banks.

Plant	Family	Perennial grasses	Upper grass
*Leymus chinensis*	Poaceae	√	√
*Stipa baicalensis*	Poaceae	√	
*Artemisia tanacetifolia*	Asteraceae		√
*Cleistogenes squarrosa*	Poaceae	√	
*Adenophora stenanthina*	Campanulaceae		√
*Thalictrum squarrosum*	Ranunculaceae		√
*Astragalus melilotoides*	Fabaceae		√
*Heteropappus altaicus*	Asteraceae		
*Potentilla nudicaulis*	Rosaceae		
*Pulsatilla turczaninovii*	Ranunculaceae		

In 2005, it was divided into five mowing regimes: 1. Complete nonmowing and nongrazing (CK); 2. Mowing events per year (C1); 3. Mowing once every two years (C2); 4. Mowing once every three years (C3); 5. Mowing once every six years (C6). Mowing timing was coordinated with the local grass-mowing season, with a stubble height of 7 cm, which is consistent with local policy.

### 2.2 Grassland germinable soil seed bank sampling

This experiment was initiated in 2005, and in 2018, 3 sample plots (each with an area of 15 m × 15 m) were randomly selected for each mowing treatment. Three 1 × 1 m quadrats were randomly selected in each sample plot, and a soil drill with a diameter of 5 cm was used for sampling in each square. Samples were taken from the 0~2 cm, 2~5 cm and 5~10 cm layers, with a total of 6 randomly selected sampling points. The soils in the same soil layer and in the same square were subsequently mixed to form a composite sample for the corresponding soil layer of the quadrat, and the soil sampling area was 6 × 2.5 × 2.5 × 3.14 = 117.75 cm^2^. The volume of each square in the 0~2 cm, 2~5 cm and 5~10 cm soil layers was 235.50 cm3, 353.25 cm3 and 588.75 cm^3^, respectively. Therefore, the samples consisted of 5 mowing treatments × 3 plots × 3 quadrats × 3 layers of soil samples = 135. The soil was placed in a shed for natural drying.

### 2.3 Seed bank germination experiments

In this study, the “soil seed bank” specifically refers to the “germinable soil seed bank”. We employed the soil sample germination method and placed the samples in a greenhouse to provide optimal germination conditions, including appropriate sunlight, moisture, and temperature. Seed germination trays (diameter, 17 cm; height, 3 cm) were selected, with vermiculite as the substrate spread evenly at the bottom of the trays. Each soil sample was then placed in the germination trays, with a soil sample thickness of 1–2 cm. Each soil sample was subsequently placed in a germination tray with a thickness of 1~2 cm. The cumulative germination curves were obtained to determine the 45-day germination rate. When the number of seeds showed minimal changes, the germination experiment was extended by an additional week to ensure that all the seeds in the soil seed bank had the opportunity to germinate. During this period, the samples were periodically watered to maintain adequate moisture levels. Species identification of the seedlings was performed through morphological, color, and odor characteristics. Species that could be identified were recorded daily for their germination counts. For those species whose seedlings could not be identified initially, their seedlings were transplanted and identified at a later stage.

### 2.4 Data analysis

#### 2.4.1 Normality test

Normality tests were separately conducted for the soil seed banks of germinable seeds, the perennial grass seed bank, the epi-phanerophyte soil seed bank, and the 5–10 cm soil seed bank (including the soil seed bank of germinable seeds, the perennial grass seed bank, and the epi-phanerophyte soil seed bank). The soil seed bank of the germinable seeds, perennial grass seed bank, and 5–10 cm soil seed bank conformed to a normal distribution, whereas the epi-phanerophyte soil seed bank data were transformed using the square root method.

#### 2.4.2 Analysis of variance

A two-way ANOVA model with replication was constructed, with the mowing regime as one influencing factor, the soil layer depth (or 5–10 cm germinable seed bank, perennial grass seed bank, and epi-phanerophyte seed bank) as another influencing factor, and three representative sample plots were used as replications to investigate the interaction effect between the mowing regime and the soil layer. When the ANOVA results were significant (considering both fitting rates), mean comparisons were conducted using Duncan’s test (significance level p<0.05). The analysis results were plotted via SigmaPlot 14.0.

## 3 Results

### 3.1 Germinable soil seed bank species under different mowing regimes and at different soil depths

**Figure 1:**
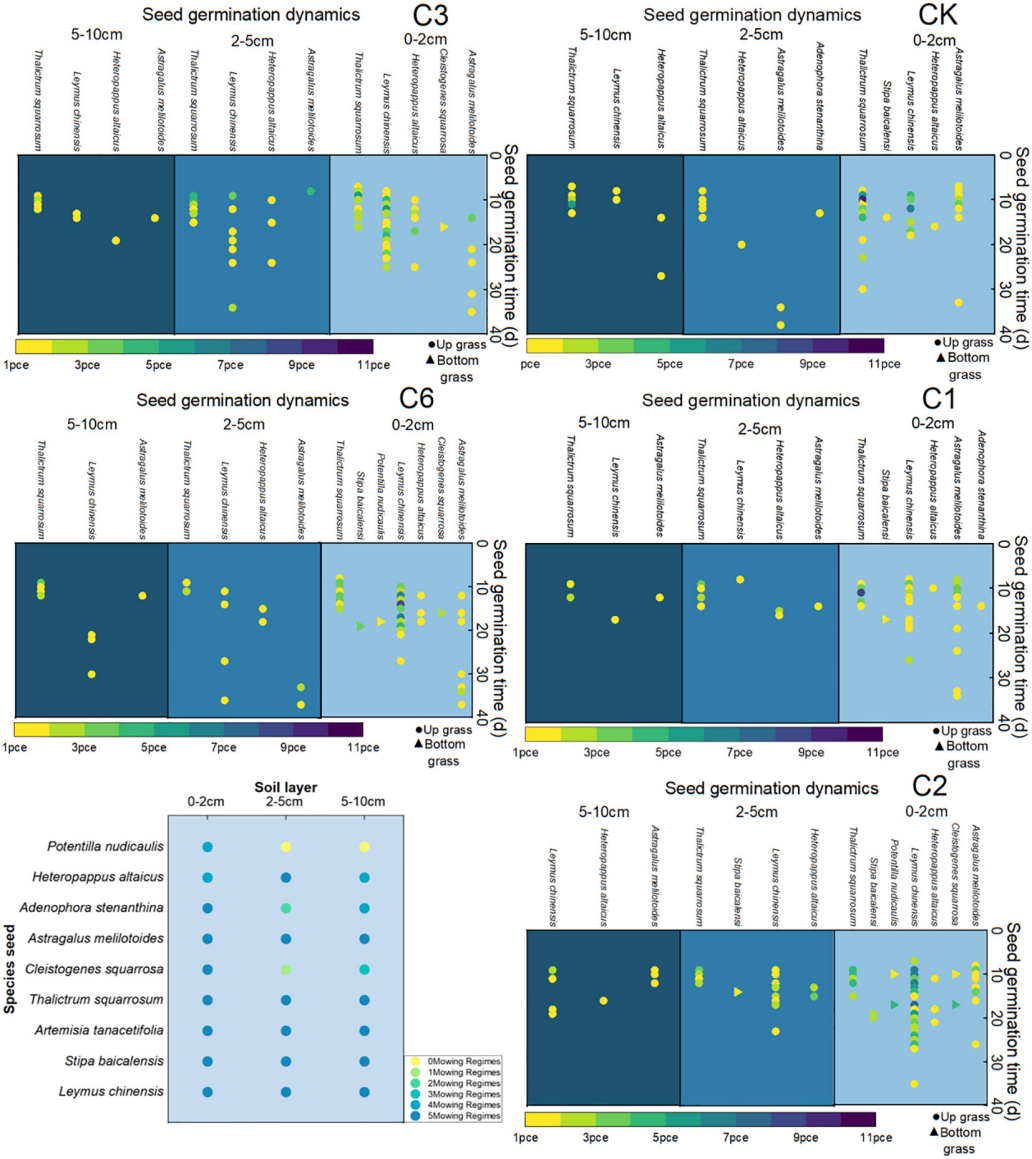
Seed dynamic germination at 45 days. Frequency of species occurrence under different mowing regimes. Note that the color gradient from yellow to purple represents an increase in the number of seed germinations. CK represents the enclosed area, C1 represents 1 mowing event per year, C2 represents 1 mowing event every 2 years, C3 represents 1 mowing event every 3 years, and C6 represents 1 mowing event every 6 years.

Under the five different mowing regimes, the seeds of *L. chinensis*, *S. baikalis*, *Artemisia schizophylla, P. chinensis, Astragalus membranaceus* and *Altai Dogflower* germinated in soils from different depths, and the seeds of the other plant populations did not germinate in soils from different depths. *S. cocophyllum* and *C. rubrum* mainly germinated in soils from depths of 0–2 cm, *S. longifolia* mainly germinated in soils from depths of 0–2 cm and 5–10 cm, and a small number of seeds germinated in soils from depths of 2–5 cm (**Figure 1**).

On the basis of 45-day germination observations, we found that the main species in the control group (CK) were *Leymus chinensis, Stipa baicalensis, Heteropappus altaicus, Astragalus melilotoides, Potentilla nudicaulis, Pulsatilla turczaninovii*, and *Thalictrum squarrosum.* The main species associated with mowing once a year are *Stipa baicalensis, Astragalus melilotoides, Heteropappus altaicus, Potentilla nudicaulis, Thalictrum squarrosum*, and *Pulsatilla turczaninovii*. The main species that mowed once every two years included *Leymus chinensis, Heteropappus altaicus, Cleistogenes squarrosa, Stipa baicalensis, Potentilla nudicaulis, Heteropappus altaicus* and *Pulsatilla turczaninovii.* The main species that mowed once every three years were *Leymus chinensis, Cleistogenes squarrosa, Pulsatilla turczaninovii, Heteropappus altaicus, Thalictrum squarrosum*, and *Astragalus melilotoides*. The main species that mowed once every six years included *Leymus chinensis, Cleistogenes squarrosa, Heteropappus altaicus, Potentilla nudicaulis, Pulsatilla turczaninovii, Stipa baicalensis* and *Thalictrum squarrosum* (**Figure 1**).

**Figure 2:**
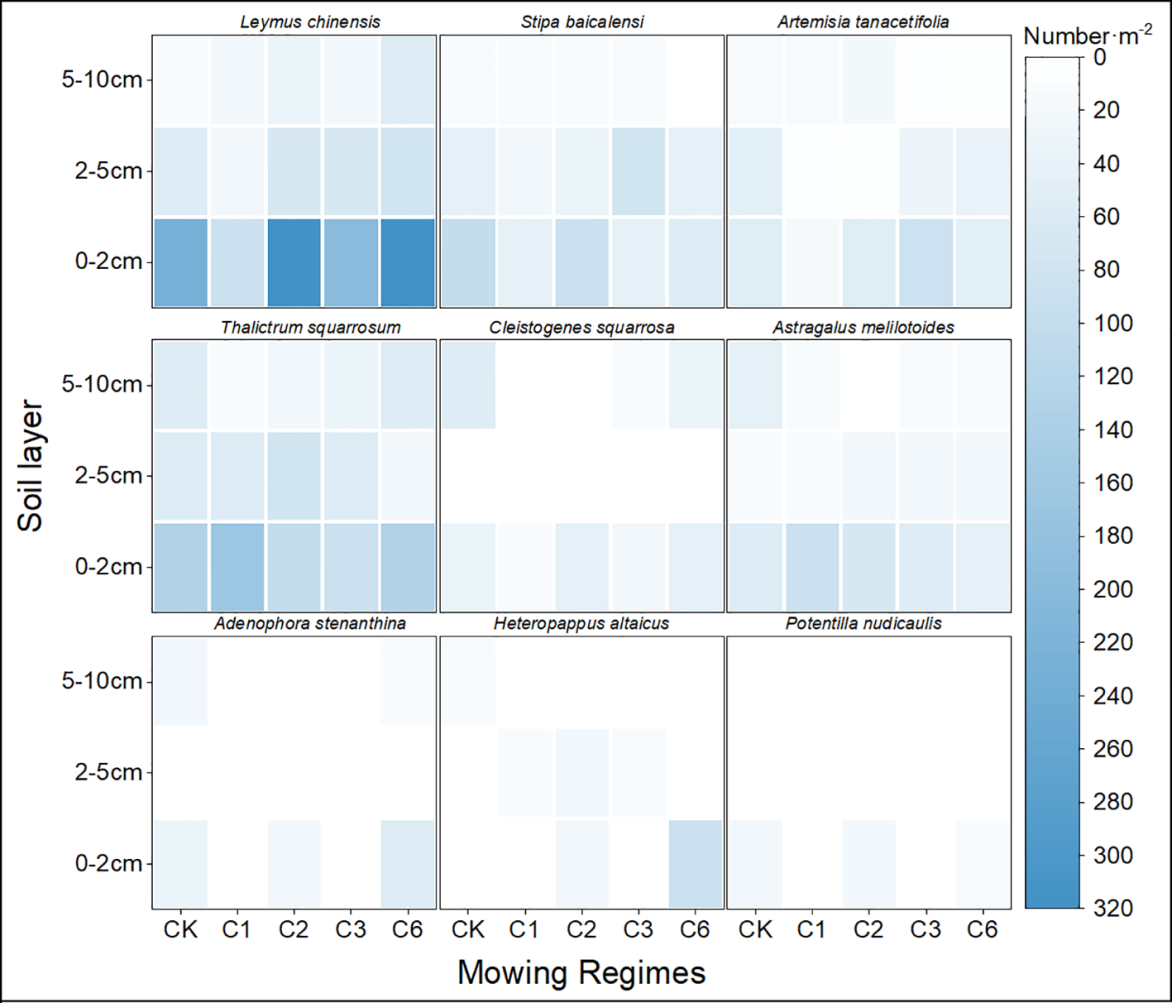
Seed density of species that germinate with respect to soil depth and mowing regime.

By comparing different mowing regimes, we found that the mowing events per year lacked the local dominant species *Leymus chinensis* and *Cleistogenes squarrosa*, both of which are high-quality forages that are highly palatable to livestock. Additionally, the quantity of seeds collected per year was the lowest, which is detrimental to the sustainable development and efficient utilization of grasslands. In contrast, both mown once every two years and mowing once every six years resulted in greater seed quantity. Compared with that of the mowing event per year, the quantity of perennial grasses significantly increased, and the abundance of the dominant species *Leymus chinensis* was notably greater than that of the other mowing regimes (**Figure 2**).

### 3.2 Effects of mowing and soil depth on the germinable soil seed bank

According to the ANOVA of the effects of mowing and soil depth on the soil seed bank (**Table 2**), soil depth had a very significant effect on the soil seed bank (**Table 2**, p<0.001), and mowing and its interaction with soil depth had a significant effect on the soil seed bank (**Table 2**, p<0.05).

**Table 2:** Analysis of variance of the effects of mowing and soil depth on the soil seed bank.

Source	DF	SS	MS	F Value	Pr>F	R-Square
Model	14	3399490.61	242820.76	21.50	<.001	0.9094
Mowing regime	4	159071.29	39767.82	3.52	0.018	
Soil layer	2	3036018.31	1518009.15	134.40	<.001	
Mowing regime ×Soil layer	8	204401.01	25550.13	2.26	0.050	
Error	30	338841.18	11294.71			
Total	44	3738331.79				

**Figure 3:**
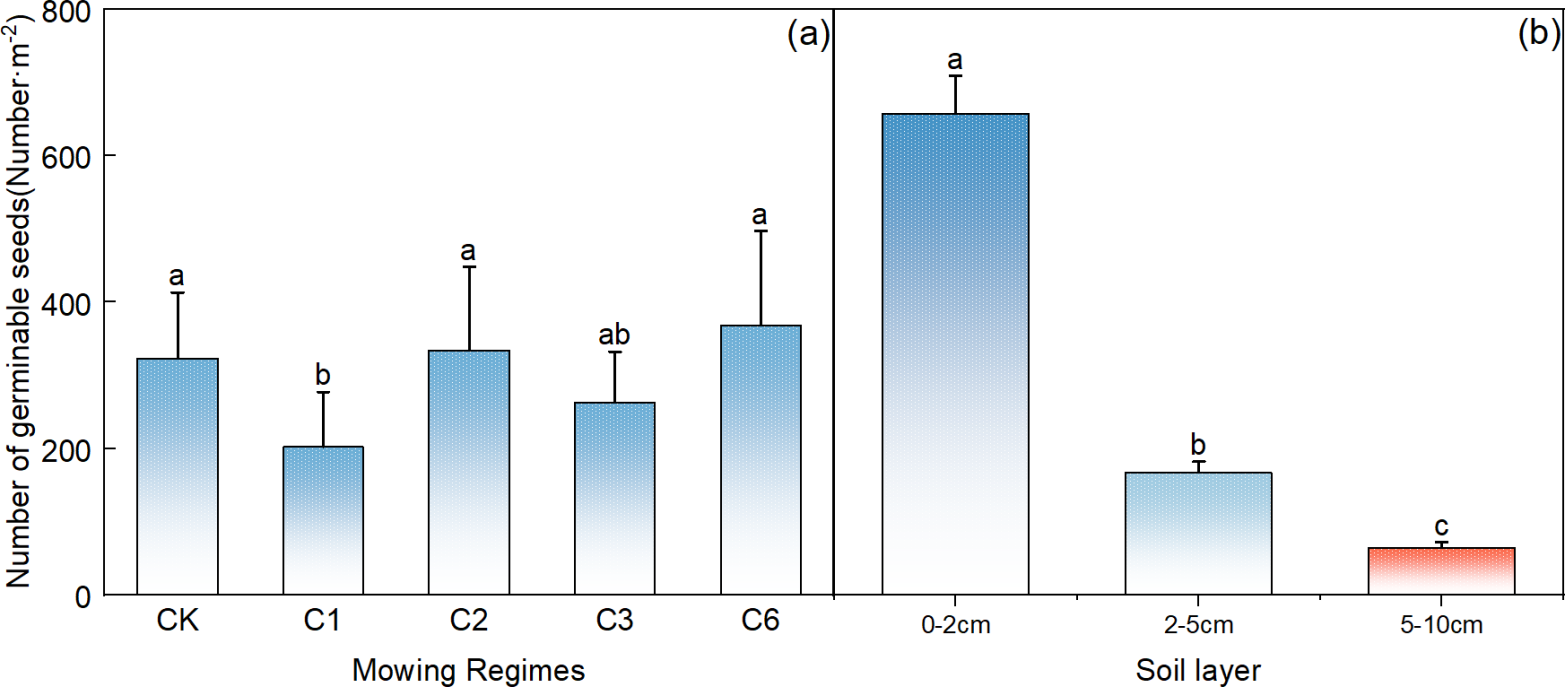
Comparative analysis of the effects of mowing regime **(A)** and soil depth **(B)** on the number of germinable seeds in the soil. CK represents the enclosed area, C1 represents 1 mowing event per year, C2 represents 1 mowing event every 2 years, C3 represents 1 mowing event every 3 years, and C6 represents 1 mowing event every 6 years. In the figure, groups with the same lowercase letters are not significantly different (P > 0.05).

Compared with that in the control area, the germination rate of the soil seed bank was significantly lower each year after the mowing event (**Figure 3A**, p<0.05). Among the different mowing regimes, seed germination was significantly greater under the mowing regime every 2 years and the mowing regime every 6 years than under the mowing regime per year (**Figure 3A**, p< 0.05). In terms of the vertical distribution of the number of germinable seeds in the soil, many germinable seeds were present in the surface layer from 0–2 cm, up to 600 seeds/m^2^, and the number decreased significantly with increasing soil depth; 0–2 cm>2–5 cm> 5–10 cm, and the number of germinable seeds at the 0–2 cm depth accounted for approximately 80% of the total (**Figure 3B**).

### 3.3 Effects of mowing and soil depth on the germinable seed bank of perennial grasses

The number of germinable seeds of perennial grasses was significantly lower in the once-a-year mowing regime (C1) (**Table 3**, p < 0.05), the total number of germinable seeds in the once-a-year mowing regime (C1) was significantly lower (**Table 3**, p< 0.05), and the other mowing systems had no significant effect (**Table 3**, p > 0.05).

**Table 3:** Analysis of variance of the effects of mowing and soil depth on the seed bank of perennial grasses.

Source	DF	SS	MS	F Value	Pr>F	R-Square
Model	14	997417.57	71244.11	36.64	<.001	0.9448
Mowing regime	4	87090.20	21772.55	11.20	<.001	
Soil layer	2	800496.45	400248.23	205.87	<.001	
Mowing regime × Soil layer	8	109830.91	13728.86	7.06	<.001	
Error	30	58325.87	1944.20			
Total	44	1055743.44				

**Figure 4:**
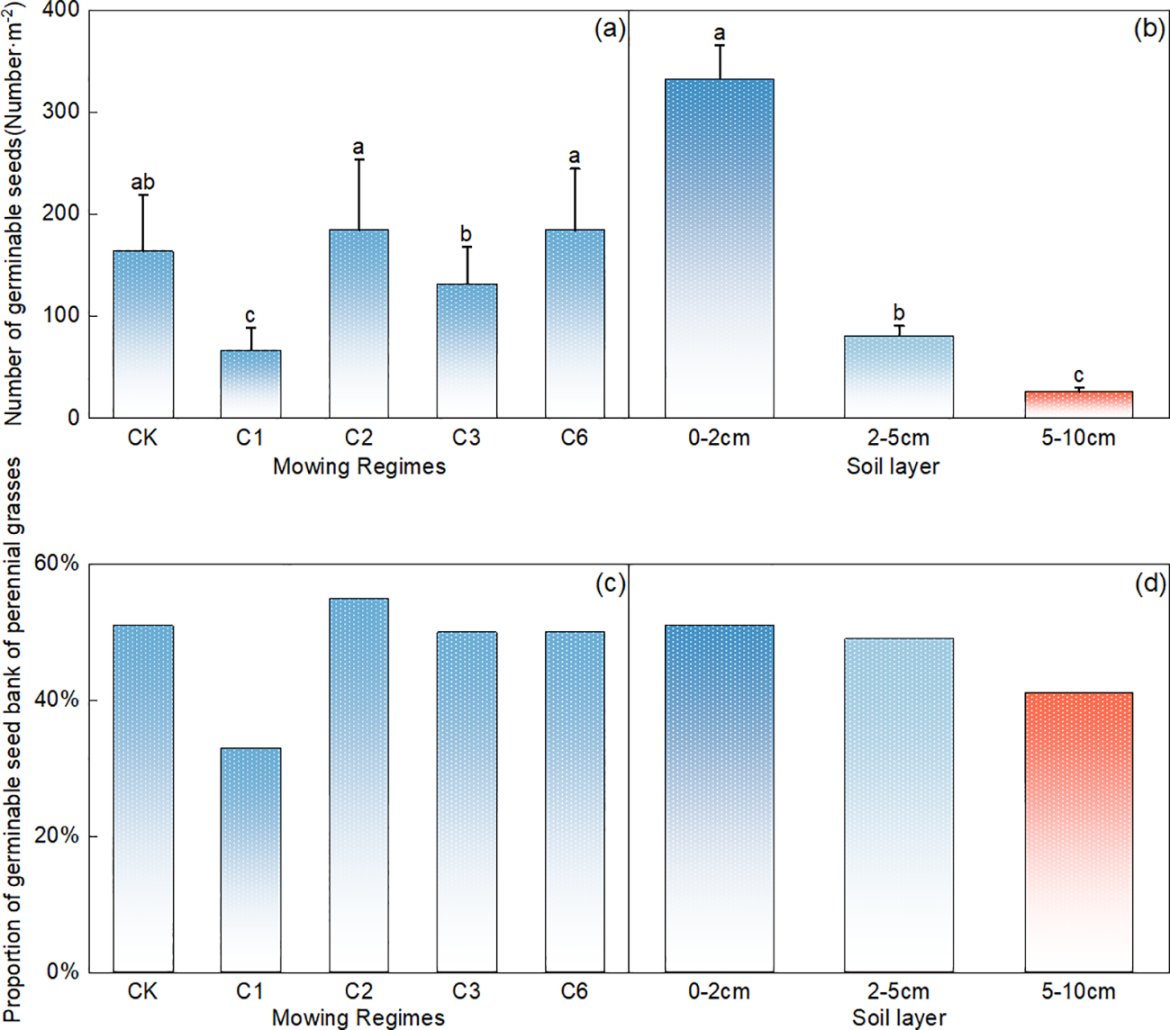
Comparative analysis of the effects of mowing regime **(A)** and soil depth **(B)** on the number of germinable perennial grass seeds. Note: Proportion of perennial grasses in the germinable seed bank under different mowing regimes **(C)** and soil depth layers **(D)**, where CK represents the control area; C1 represents 1 mowing event per year; C2 represents 1 mowing event every 2 years; C3 represents 1 mowing event every 3 years; C6 represents 1 mowing event every 6 years. In the figure, groups with the same lowercase letters are not significantly different (P > 0.05).

Compared with the other mowing systems, mowing events per year resulted in a significantly lower number of germinable perennial grasses (**Figure 4A**, p<0.05). The number of germinable perennial grasses decreased significantly with increasing soil depth (**Figure 4B**, p<0.05), with 0–2 cm > 2–5 cm > 5–10 cm, and the number of germinable perennial grasses in the 0–2 cm soil layer accounted for 78% of the total germinable seeds (**Figure 4B**).

Compared with that in the control area, the proportion of perennial grasses in the mowing event per year decreased significantly, whereas a slight increase was observed in the once-every two-year mowing treatment (**Figure 4C**). Additionally, across the different soil layers, the number of perennial grass seeds tended to decrease with increasing depth (**Figure 4D**).

### 3.4 Effects of mowing system and soil depth on the number of germinating seeds in upper grass

The analysis of variance of the effects of the mowing regime and soil depth on the number of germinable seeds of upper grasses revealed that mowing had a significant effect on the seed bank of upper grasses (**Table 4**, p<0.05) and that soil depth had a very significant effect on the seed bank of upper grasses (**Table 4**, p<0.001), but the interaction between the two had no significant effect on the seed germination of upper grasses (**Table 4**, p=0.641).

**Table 4:** Analysis of variance of the effects of mowing and soil depth on the number of upper grass seeds.

Source	DF	SS	MS	F Value	Pr>F	R-Square
Model	14	2103.23	150.23	21.92	<.001	0.9109
Mowing regime	4	77.46	19.36	2.82	0.042	
Soil layer	2	1984.18	992.09	144.73	<.001	
Mowing regime × Soil layer	8	41.60	5.20	0.76	0.641	
Error	30	205.64	6.85			
Total	44	2308.87				

**Figure 5:**
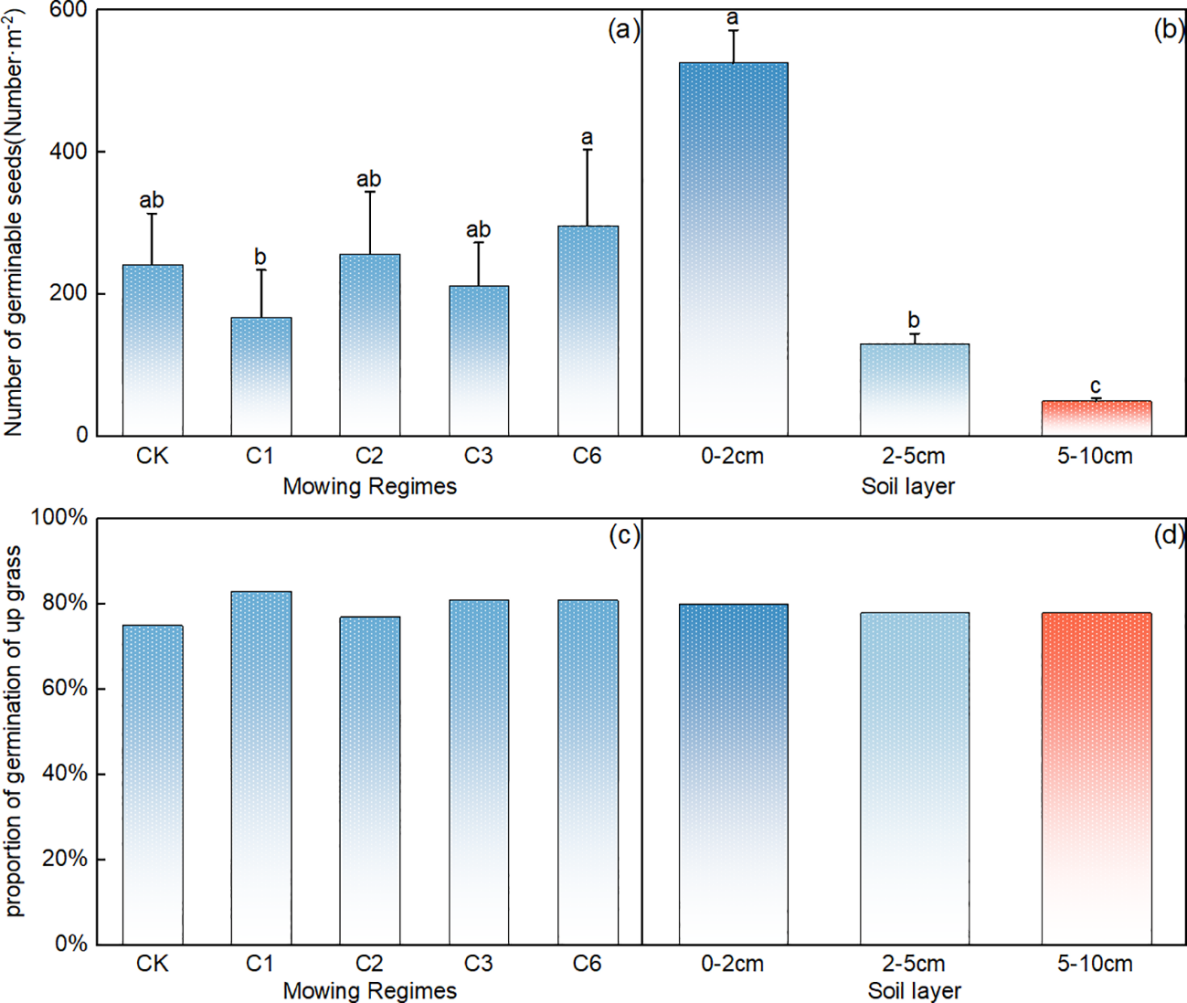
Comparative analysis of the effects of mowing regime **(A)** and soil depth **(B)** on the number of germinable upper grass seeds; note: Proportion of upper grasses in the seed bank under different mowing regimes **(C)** and various soil depths **(D)**. CK represents the control area, C1 represents 1 mowing event per year, C2 represents 1 mowing event every 2 years, C3 represents 1 mowing event every 3 years, and C6 represents 1 mowing event every 6 years. In the figure, groups with the same lowercase letters are not significantly different (P > 0.05).

Compared with that in the control area, the number of germinable seeds of upper grasses decreased the most under the once-a-year mowing regime (**Figure 5A**), the number of germinable seeds of upper grasses significantly increased annually after the mowing event (**Figure 5A**, p < 0.05), and there was no significant difference among the other mowing regimes (**Figure 5A**, p>0.05). The number of germinable grass seeds decreased significantly with increasing soil depth (p<0.05), with a 0–2 cm>2–5 cm>5–10 cm trend, and the number of germinable seeds in the top 0–2 cm of soil accounted for 72% of the total number of germinable grass seeds (**Figure 5B**).

The proportion of dominant grass seeds remained above 60% across all the treatments. Compared with that in the control area, there was a slight increase in the proportion of dominant grass under the mowing event per year (**Figure 5C**). Furthermore, the proportion of dominant grass seeds across different soil layers also exceeded 60% (**Figure 5D**).

### 3.5 Changes in the 5–10 cm depth soil seed germinable bank

The analysis of variance revealed that mowing and the components of the soil seed bank had very significant effects on the number of seeds that germinated in the 5–10 cm depth soil bank (**Table 5**, p<0.001), but the effect of the interaction between the two variables was not significant (**Table 5**, p=0.726).

**Table 5:** Analysis of variance of the effects of mowing and soil depth on the number of germinable seeds in the 5–10 cm depth soil seed bank.

Source	DF	SS	MS	F Value	Pr>F	R-Square
Model	14	18646.48	1331.89	5.52	<.001	0.7202
Mowing regime	4	6808.70	1702.18	7.05	0.001	
Germinable soil seed bank components	2	10573.86	5286.93	21.89	<.001	
Mowing regime ×Germinable soil seed bank components	8	1263.91	157.99	0.65	0.726	
Error	30	7244.08	241.47			
Total	44	25890.57				

**Figure 6:**
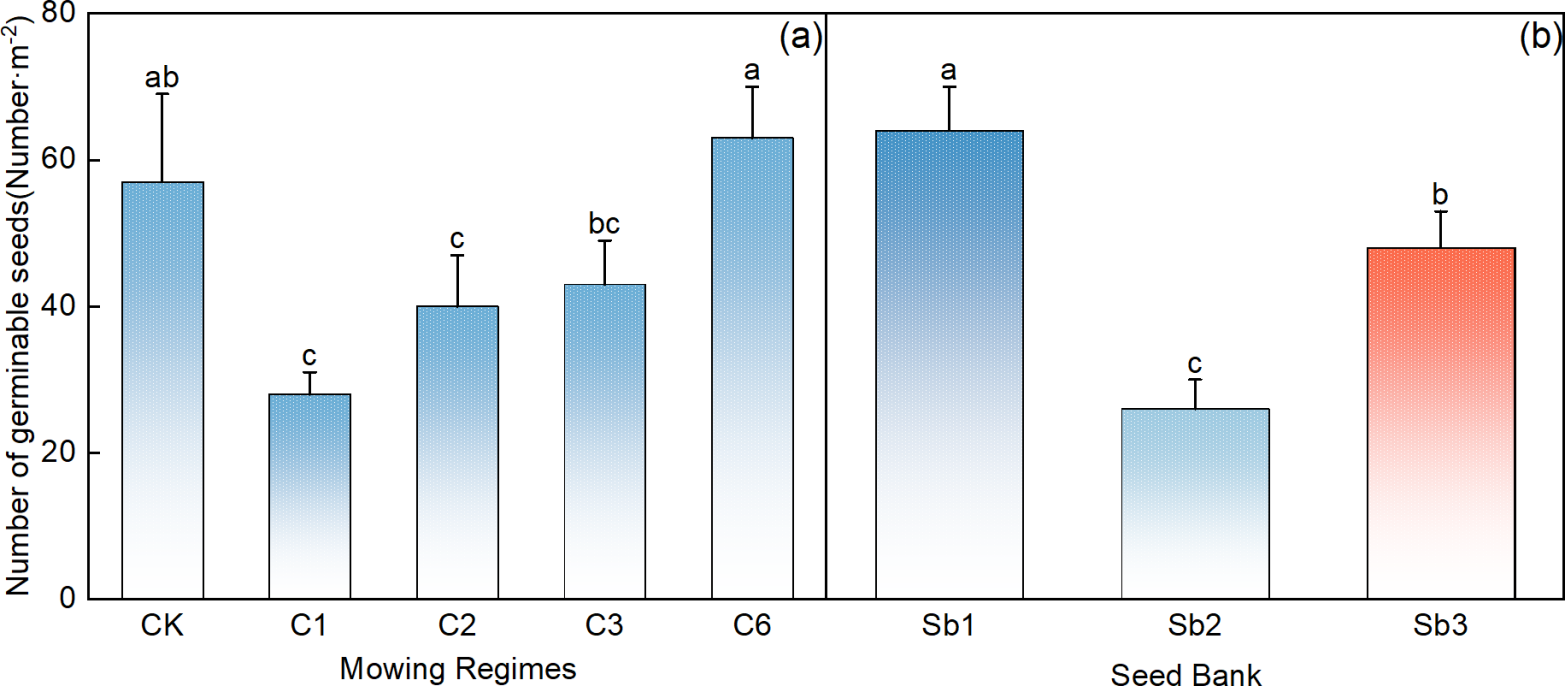
Comparative analysis of the effects of mowing regimes **(A)** on the 5–10 cm depth **(B)** soil seed bank. CK represents the control area, C1 represents 1 mowing event every year, C2 represents 1 mowing event every 2 years, C3 represents 1 mowing event every 3 years, C6 represents 1 mowing event every 6 years, and Sb1-Sb3 represent the seed banks of the 5–10 cm soil, perennial grasses, and upper grasses. In the figure, groups with the same lowercase letters are not significantly different (P > 0.05).

Compared with that in the control area, the number of germinable seeds in the 5–10 cm soil layer was significantly lower in the mowing event per year and in the once-every-2-year mowing regime (**Figure 6A**, p< 0.05). Among the 5–10 cm depth soil germinable seeds, the number of seeds that could germinate in the 5–10 cm soil layer was greatest in the treatment involving mowing every 6 years (**Figure 6A**), in which the rate of germination was significantly greater than that under the other mowing regimes (**Figure 6A**, p<0.05). There were significant differences in the number of germinable seeds in the 5–10 cm soil, perennial grass, and upper grass seed banks (**Figure 6B**, p<0.05), with the largest number found in the total soil seed bank, followed by that in the upper grass seed bank, and the lowest number was found in the perennial grass seed bank.

## 4 Discussion

### 4.1 Effects of mowing on the germinable soil seed bank

In this work, the effects of different mowing regimes on soil seed banks in different soil layers were studied. The results revealed that the number of seeds in the soil seed bank significantly decreased under the different mowing regimes. The number of seeds in the 0–2 cm soil layer was the greatest, accounting for approximately 80% of the total, followed by the number of seeds in the 2–5 cm soil layer, and the lowest number of germinable seeds was found in the 5–10 cm soil layer (**Figure 2B**).

The results revealed that the seeds in the soil seed bank were concentrated mainly in the soil surface layer (0–2 cm), and the seed bank density decreased significantly with increasing depth, which was consistent with the vertical distribution of the seed bank reported by Tóth (2023) (Tóth et al., 2022).

Mowing affects aboveground plant communities, subsequently influencing changes in the soil surface and biota, as well as soil physical and chemical properties (Yuan et al., 2011). Mowing regimes can alter plant reproductive strategies. A postmowing reduction in aboveground biomass affects light absorption and CO_2_ uptake. For example, *the Leymus* chinensis leaf photosynthesis rate increases by 20% within a week after mowing to support rapid growth. However, continuous annual mowing allocates most of the energy to physiological compensation and little energy to reproduction, potentially explaining the significant decline in soil seed bank density with mowing events per year (Sugimoto et al., 2019; Piseddu et al., 2021). Annual mowing removes substantial aboveground biomass, altering ground cover and reducing litter, thereby increasing surface light intensity and the daily temperature range, all of which affect seed germination and community composition, as observed in the comparisons between C1 and C6, where the viable seed count of C1 was the lowest (**Figure 2**). Li (2024) reported that the litter decomposition rate decreases with temperature, affecting ecosystem turnover and nutrient release processes, intensifying intraspecific and interspecific competition among seeds, and influencing seed survival and germination rates (Liu et al., 2021; Mašková et al., 2022). Other mowing regimes (CK, C2, C3, and C6) with increased intervals provide plants with ample time for physiological compensation and reproduction, increasing ground cover, reducing diurnal soil temperature fluctuations, increasing soil nutrients, increasing seed survival and germination rates, and creating more favorable conditions for seed germination.

Compared with 1 mowing event per year, extending the mowing cycle (mowing once every two years, mowing once every three years, and mowing once every six years) is more conducive to increasing the number of germinable seeds in soils.

### 4.2 Effects of mowing on perennial grasses

As a dominant species in temperate *L. chinensis* grasslands, the main perennial grasses in the study area are *L. chinensis, S. baikal* and *S. columbia*, which constitute important populations in the Hulunbuir natural grassland in Inner Mongolia, China. The results revealed that, among the mowing regimes, the overall variation was the same in the soil seed bank; perennial grass seeds were concentrated mainly in the 0–2 cm soil layer, the germinable seed count in the 2–5 cm soil layer ranked second, and the 5–10 cm soil layer had the lowest number of germinating seeds.

The perennial grass seed bank is a key factor in restoring grassland productivity (Guo et al., 2023). Successive mowing disrupts perennial grass reproduction, reducing the chance for growth. Guo (2023) noted that high perennial grass seed density is a key factor for the success of spontaneous grassland restoration (Guo et al., 2023). Additionally, Zhao et al. reported that the supply of water and nitrogen significantly increases the seed yield of *Leymus chinensis*, and Yang (1989) noted that water and nitrogen affect the development of seed-forming organs. Compared with other mowing regimes, continuous mowing results in relatively less litter, potentially leading to soil water loss and insufficient nutrient provision from litter decomposition, thereby intensifying intraspecific and interspecific competition. This likely contributes to the lowest content of soil seed banks in the once-yearly mowing regime, whereas in the other mowing regimes (CK, C2, C3, and C6), plants have ample time for self-repair and reproduction (Dong et al., 2020; Jian-cai et al., 2022). The dominant species in the soil seed bank of degraded grasslands are annual and perennial weeds (Guo et al., 2023); therefore, once-a-year mowing is not conducive to the survival and reproduction of perennial grasses and their seed banks in meadow steppes. Once-a-year mowing is very common and does not support sustainable use, whereas in other regimes, such as mowing every two years, good habitats and seed banks are formed, maintaining long-term sustainability in temperate *L. chinensis* meadow grassland communities.

### 4.3 Effects of mowing on the germinable seed bank of upper grasses

Our investigation revealed congruent germination patterns between the upper grass soil seed banks and the overall seed bank dynamics, with both predominantly accumulating at the soil surface. Under the regime of annual mowing, a significant depletion of the soil seed bank content was observed in the region, where the upper grass consisted primarily of *Leymus chinensis, Artemisia tanacetifolia, Astragalus melilotoides, Thalictrum squarrosum*, and *Adenophora stenanthina.*

Upper grasses are characterized by reproductive shoots and long vegetative shoots, with the above-ground portion of the plant being notably tall, and their seed formation organs are susceptible to mowing interference (Jian-cai et al., 2022; Yan et al., 2023). The impact of annual mowing on the soil seed bank can be explained by the theoretical framework of energy redistribution within the soil seed bank under mowing regimes. Individual plants exhibit variability in their phenological phases due to differences in seed germination positions and soil nutrient availability, implying that not all seeds complete their reproductive phases by the time of harvest, contributing to potential losses in the seed bank.

Given that upper grasses, including *L. chinensis* and *A. tanacetifolia*, are the dominant species in the area and are numerically superior, they present greater reproductive opportunities and consequently dominate the seed bank content in the soil (Yan et al., 2023). Seed bank dynamics play a pivotal role in determining overall soil seed bank changes, with sustained seed bank germination and establishment being crucial for the restoration and maintenance of grassland health (Tóth et al., 2022).

Light is essential for plant growth and reproduction, as plants harness light via photosynthesis to convert CO_2_ and water into the energy required for growth and reproduction while also producing oxygen. In early succession stages, lower stratum light allocation is generally greater within grassland plant communities (Connell and Slatyer, 1977). During succession, upper grasses increasingly dominate the region due to their large above-ground structures, impeding sunlight to shorter herbaceous plants and thereby reducing their biomass. Nonetheless, the soil seed bank typically encompasses seeds of all flora from the initial succession to the current stage. Light is also a vital factor for the germination of certain seeds (Fenner and Thompson, 2005; Hautier et al., 2009). Studies indicate that, compared with taller plant species, lower stratum plant seed germination often requires more light, which can germinate without light (Milberg et al., 2000; Bu et al., 2016).

With prolonged succession, the dominance of tall upper grass enhances grassland quality and productivity, increases soil fertility, and provides quality feed for livestock (Zhang et al., 2022). Annual mowing results in substantial losses in the soil seed bank, leading to their decline as dominant species and allowing more space for weed proliferation. This imbalance cannot effectively replenish the soil seed bank, ultimately impacting the overall seed bank dynamics and consequently the regeneration of plant communities in subsequent years, reducing community biomass and enabling weeds to dominate, thereby degrading pasture quality.

Strategically, avoiding annual mowing and adopting biennial mowing intervals would be more suitable, as shorter intervals maintain and effectively replenish the soil seed bank, promoting the sustainable utilization of warm temperate meadow grasslands.

## 5 Conclusions

Mowing significantly affected the soil seed bank, and each mowing event significantly reduced the number of soil seeds, perennial grass-germinable seeds, and germinable seeds of upper grasses. With increasing soil depth, the number of germinable seeds in the soil, perennial grass-germinable seeds and upper grass-germinable seeds decreased significantly, and most of the germinable seeds were concentrated in the surface soil layer (0–2 cm). Mowing every 2 years and mowing every 6 years can increase the number of germinable seeds in the soil, which can effectively protect and promote the health and sustainability of grassland ecosystems.

## Data Availability

The raw data supporting the conclusions of this article will be made available by the authors, without undue reservation.
